# Inhibitory Effect of Ascorbic Acid on *in vitro* Enzymatic Digestion of Raw and Cooked Starches

**DOI:** 10.3389/fnut.2021.758367

**Published:** 2021-11-26

**Authors:** Jiayue Guo, Alyssa Gutierrez, Libo Tan, Lingyan Kong

**Affiliations:** ^1^Department of Human Nutrition and Hospitality Management, The University of Alabama, Tuscaloosa, AL, United States; ^2^Department of Biological Sciences, The University of Alabama, Tuscaloosa, AL, United States

**Keywords:** high amylose maize starch, potato starch, ascorbic acid, cooking, simulated *in vitro* digestion

## Abstract

Ascorbic acid, also known as vitamin C, was previously reported to inhibit the activity of pancreatic α-amylase, the primary digestive enzyme for starch. A major implication of such inhibition is a slowed rate of starch digestion into glucose, which thereby reduces postprandial hyperglycemia. The aim of this study was to explore the inhibitory effects of ascorbic acid at various concentrations on the *in vitro* digestion of high amylose maize starch (HAMS) and potato starch (PS) in both raw and cooked conditions. Resistant starch (RS) content, defined as the starch that remained after 4 h of simulated *in vitro* enzymatic digestion, was measured for the starch samples. Upon the addition of ascorbic acid, the RS contents increased in both raw and cooked starches. Cooking significantly reduced the RS contents as compared to raw starches, and less increase in RS was observed with the addition of ascorbic acid. The inhibitory effect of ascorbic acid on the digestion of raw starches showed a dose-dependent trend until it reached the maximum extent of inhibition. At the concentrations of 12.5 and 18.75 mg/mL, ascorbic acid exhibited the most potent inhibitory effect on the *in vitro* starch digestion in raw and cooked conditions, respectively. Overall, our results strongly indicate that ascorbic acid may function as a glycemic modulatory agent beyond other important functions, and its effects persist upon cooking with certain concentrations applied.

## Introduction

As one of the most common chronic diseases worldwide, diabetes mellitus is a major risk factor for cardiovascular diseases and is associated with an increased rate of morbidity and mortality (1). It is characterized by chronic hyperglycemia, which involves many alterations at the vascular tissue that accelerates the pathogenesis of diabetic complications (2). Among the determinants for glucose metabolism, diet plays an important role in the development of hyperglycemia, as excessive ingestion of calorie-dense and easily digestible foods can cause abnormal spikes in postprandial blood glucose level (3, 4). Since starch is the primary energy source in human diet, retarding starch digestion and glucose absorption could serve as an effective way for the prevention and treatment of hyperglycemia and related metabolic diseases (5–7). Accordingly, some antidiabetic drugs, such as acarbose, have been used to retard starch digestion through inhibiting the activity of digestive enzymes (8). Nevertheless, some drugs are reported to have certain side effects, such as hepatotoxicity, gastrointestinal disturbances and diarrhea (9). Therefore, food-based strategies are of great interest as they may have less side-effects and carry additional health benefits. As a group of dietary compounds with beneficial health effects, phenolic compounds have been proposed as natural inhibitors of human digestive enzymes, including α-amylase, α-glucosidase, and lipase, etc. One of the well-known compounds is ascorbic acid, which has demonstrated the ability to inhibit starch digestive enzymes (10, 11) and therefore may function as a glycemic modulatory agent.

Ascorbic acid, commonly known as vitamin C, is a water-soluble micronutrient essential for the proper functioning of the body. It can be found in many foods, particularly fruits and vegetables such as citrus fruit, broccoli, and spinach. It is required for the formation and maintenance of connective tissues, and serves as a potent antioxidant which protects the body from harmful free radicals (12). Since it is water-soluble, excess ascorbic acid is easily excreted in urine and rarely accumulates to toxic levels. Such property makes the utilization of ascorbic acid advantageous over medications that may cause adverse symptoms at high doses. Beyond being required for the aforementioned essential metabolic activities, ascorbic acid has also been shown to inhibit the activity of pancreatic α-amylase, a digestive enzyme that plays the major role in breaking down starch into glucose, via non-competitive antagonism (11). Although the mechanism of such enzymatic inhibition was well-investigated, the effect of ascorbic acid was only examined in an α-amylase assay over the brief course of 30 min (11), which is only a fraction of the total digestive time of starch in the small intestinal tract. Moreover, few studies have evaluated the inhibitory effects of ascorbic acid with various concentrations using simulated *in vitro* digestion assays, therefore raising concern regarding the dose responses and how this may translate to real-life applications.

The objective of this study was to further investigate the inhibitory effect of ascorbic acid on starch digestion as well as to explore its general applicability as a glycemic modulatory agent. To simulate the digestion process in the small intestine, mixtures of starch and ascorbic acid were subjected to 4 h of *in vitro* digestion. The amount of starch that remained undigested after the 4-h period was defined as the resistant starch (RS) content (13). Different concentrations of ascorbic acid were used to determine whether the inhibitory effect is dose dependent. In addition, starches were subjected to cooking prior to digestion to evaluate the practicality of ascorbic acid as a potential glycemic modulatory agent.

## Materials and Methods

### Materials

High amylose maize starch (HAMS, Hylon VII) was kindly provided by Ingredion (Bridgewater, NJ, USA). Potato starch (PS, S2004) and ascorbic acid were purchased from Sigma-Aldrich Inc. (St. Louis, MO, USA). Digestible starch and resistant starch assay kit (K-DSTRS) was obtained from Megazyme (Wicklow, Ireland). Ethanol was purchased from VWR International (Radnor, PA, USA).

### *In vitro* Digestion

The *in vitro* starch digestion was conducted according to the method by the Megazyme resistant starch assay kit with slight modifications. The enzyme solution, containing pancreatic α-amylase and α-amyloglucosidase (0.8 and 0.34 KU/mL, respectively), was prepared immediately before use. Cooked starch was prepared by boiling starch in a 100°C water bath for 20 min and allowed to cool to room temperature (20°C) before *in vitro* digestion. Starch (100 mg) alone and starch mixed with 12.5, 25, 50, or 75 mg of ascorbic acid were weighed into 20 mL round-bottom test tubes. A test tube with no starch and inhibitors was used as the blank. An amount of 3.5 mL of 50 mM sodium maleate buffer was added into each tube, and the reaction suspensions were mixed and placed in a water bath at 37°C for 5 min to equilibrate. Enzyme solution (0.5 mL) was added into each tube at a certain time interval with accurate timing for further sampling. The test tubes were capped and placed into a shaking water bath at 37°C and 170 strokes per min. After incubating for 4 h, 4.0 mL of 95% (v/v) ethanol was added to each tube and was mixed vigorously. The samples were centrifuged (3,600 g, 10 min) and the supernatant was decanted. The pellet was resuspended in 8 mL of 50% (v/v) ethanol and vigorously mixed. The centrifugation, washing, and decanting steps were then repeated twice, and the remaining pellet was used for the resistant starch measurements.

### Resistant Starch Content

The RS content in starch samples was determined following the resistant starch assay procedure using the digestible starch and resistant starch assay kit (K-DSTRS) (14), with slight modifications. The remaining pellet obtained from the last step was resuspended in 2 mL of cold 1.7 M NaOH by stirring for 20 min in an ice bath, and then added with 8 mL of 1.0 M sodium acetate buffer and 0.1 mL of α-amyloglucosidase (3,300 U/mL). The tubes were mixed well and placed into a 50°C water bath for 30 min. For samples containing >10% RS content, the contents of the tubes were transferred and volumes adjusted to 100 mL in a volumetric flask with water. Aliquots of the diluted solutions were centrifuged (17,900 g, 5 min). For samples containing <10% RS content, aliquots of solutions (no dilution) were centrifuged directly (17,900 g, 5 min). In duplicates, aliquots of 30 μL from the diluted or undiluted supernatants were measured for the glucose concentrations using the glucose oxidase-peroxidase (GOPOD) method (14). The reagent blank was prepared using 30 μL of 100 mM sodium acetate buffer, and the glucose standard was prepared in duplicates using 30 μL of D-glucose (1 mg/mL). The absorbance was measured at 510 nm against the reagent blank. The RS contents were obtained using the appropriate MegaCalc^TM^ Excel^®^ Calculator.

### Statistical Analysis

All experiments were conducted in duplicates. Data were analyzed by one-way analysis of variance (one-way ANOVA) followed by Tukey's multiple comparison test using the OriginPro software (OriginLab, Northampton, MA, USA). The letters a, b, c, and d indicate statistically significant differences, *p* < 0.05 (a > b > c > d).

## Results and Discussion

### Dose-Dependent Effect of Ascorbic Acid on Inhibiting Raw Starch Digestion

**Figure 1:**
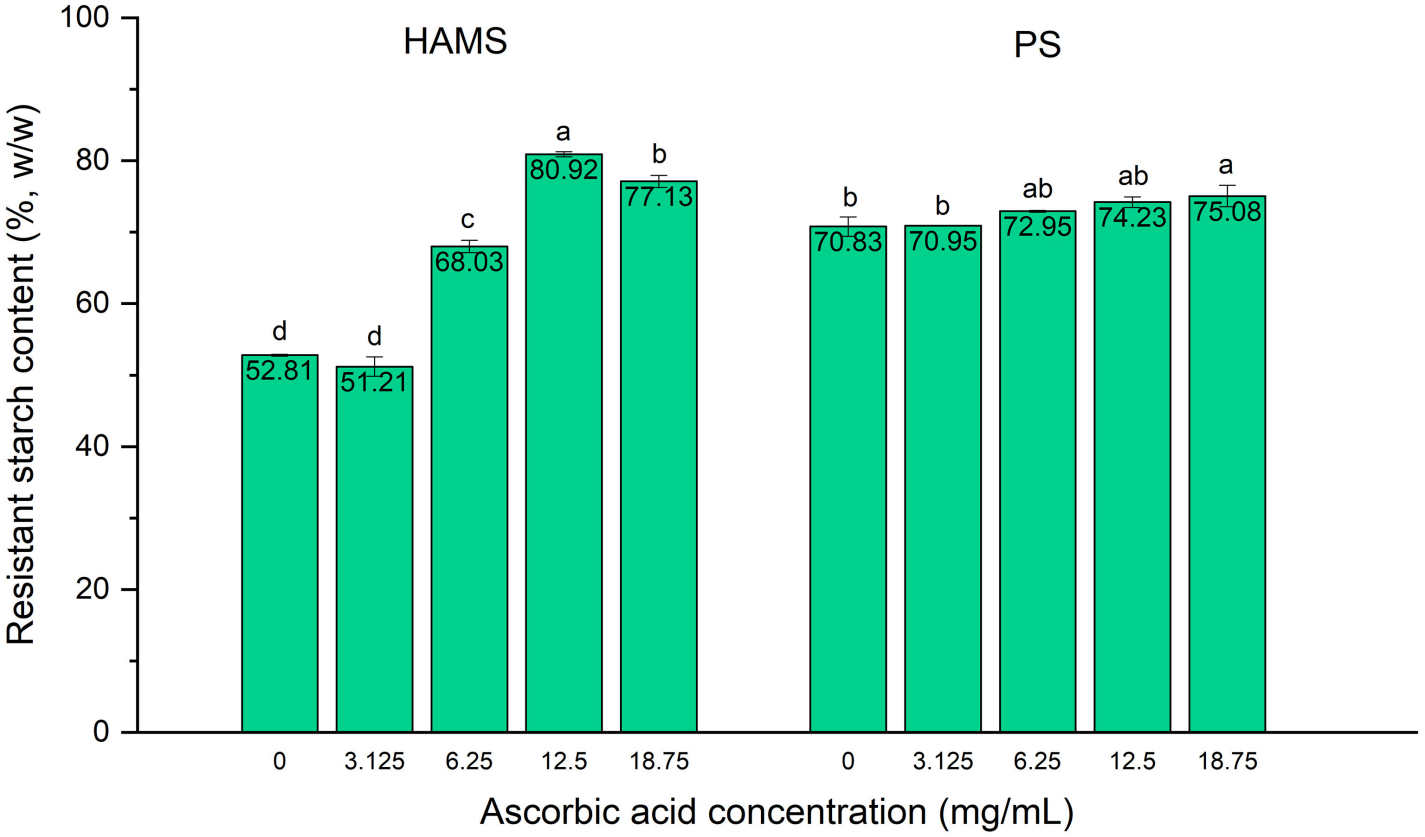
Resistant starch contents in high amylose maize starch (HAMS) and potato starch (PS), presented as a proportion of total starch, with the presence of ascorbic acid at various concentrations. Error bars show standard deviation; *n* = 2. Significant differences among treatments of differing ascorbic acid concentrations are denoted by different letters (a > b > c > d, *p* < 0.05).

The potential inhibitory effect of ascorbic acid on the *in vitro* starch digestion rate was first explored on two starches (HAMS and PS) in their raw form. To better understand its applicability as an inhibitor of starch digestion, four different concentrations (3.125, 6.25, 12.5, and 18.75 mg/mL) were used to determine the most effective level of ascorbic acid that could retard starch digestion to the greatest extent. As shown in Figure 1, the RS content of raw HAMS was 52.81%, which is very close to previous findings in HAMS, e.g., the reported 46.9% in Hi-Maize 260 starch (15), and the reference value, 47.4% in HAMS Hylon VII, provided by Megazyme (14). Upon the addition of ascorbic acid, the RS content in HAMS exhibited significant increase (*p* < 0.05) in response to all tested concentrations except for 3.125 mg/mL. As the concentration of ascorbic acid increased up to 12.5 mg/mL, the RS content continued to increase, showing a dose-dependent effect of ascorbic acid (Figure 1). However, increase of the ascorbic acid concentration to 18.75 mg/mL did not result in further increases in the RS content, demonstrating a saturation effect. Similar results were observed in PS, where a trend for dose-dependent increase in the RS content was visible as ascorbic acid concentration increased from 3.125 to 18.75 mg/mL, although only the concentration of 18.75 mg/mL resulted in a statistically significant increase.

As previously mentioned, such inhibitory effect of ascorbic acid on starch digestion is primarily due to the inhibition against α-amylases. The underlying mechanism could be owing to the hydroxyl groups present in the ascorbic acid molecules, which may be crucial in binding to pancreatic α-amylase, leading to the inhibitory activity (11). When the concentration of ascorbic acid increases, multiple ascorbic acid molecules can contribute more hydroxyl groups in forming hydrogen bonds with amino acid residues in the α-amylase binding sites, which may explain the dose-dependent inhibitory effect. Besides, the changed pH by ascorbic acid in the reactant was also considered for such inhibitory effect. We tested the pH values of the original maleate buffer and with the addition of ascorbic acid of different concentrations, and found that the pH of the buffer solution reduced from 6.01 to 5.33 (with 3.125 mg/mL ascorbic acid), 4.56 (with 6.25 mg/mL), 3.91 (with 12.5 mg/mL), and 3.65 (with 25 mg/mL) with increasing ascorbic acid concentrations, respectively. As the optimal pH for α-amylase and amyloglucosidase activity was in the range of 4.5–7.2 and 4.2–5.5, respectively (17), addition of ascorbic acid with concentrations up to 12.5 mg/mL would create an acidic environment with the pH out of the optimal ranges of the starch digestive enzymes, which is another possible mechanism underlying the inhibitory effect of ascorbic acid against starch digestion. Consequently, our findings suggest similar result as reported by Borah et al. (11), who found that the inhibitory effect of ascorbic acid against human pancreatic α-amylase is comparable to that of the reference inhibitor, i.e., acarbose, marking its strong potential as a starch digestion inhibitor and glycemic response modulator.

In this study, four different doses of ascorbic acid were applied, which were 3.125, 6.25, 12.5, and 18.75 mg/mL. Such doses were selected based on our previous finding that the addition of 25 mg of ascorbyl palmitate was effective enough to significantly increase the RS content in the same amount of starch (100 mg) (16). Based on this, to test the inhibitory effect in response to dose, ascorbic acid with amounts of 12.5, 25, 50, and 75 mg were added to 100 mg of starch prior to the *in vitro* digestion. As the total volume of the solvent was 4 mL, the concentrations of the added ascorbic acid correspond to 3.125, 6.25, 12.5, and 18.75 mg/mL, respectively. In addition, such doses are practical and applicable to incorporate into daily diet without causing any toxic effect. The absorption of ascorbic acid in small intestine is tightly regulated and peaks at ~162 mg/day (18). Since ascorbic acid is water-soluble and is easily excreted in urine, it rarely accumulates to toxic levels in tissues and plasma. At high intakes, the most common symptoms are gastrointestinal issues resulting from the osmotic effect of high concentrations of unabsorbed ascorbic acid, such as diarrhea and nausea (18). In the small intestinal tract, the total fluid volume reaches a maximum of ~94 mL (19). Using the optimal concentration of ascorbic acid at 12.5 mg/mL, the amount of ascorbic acid required (~1,175 mg) to saturate the peak fluid volume is still substantially below the tolerable upper intake level of ascorbic acid for adults at 2,000 mg/day (18). Even after compensating for ascorbic acid that is ultimately absorbed in the small intestine, the amount of ascorbic acid required to reach the maximum inhibitory activity is unlikely to be toxic in human applications. Meanwhile, it is practical to achieve such optimal dose of vitamin C via daily dietary intake or supplementation. For dietary sources, one serving of fruit or vegetable could contain up to 400 mg of vitamin C, and supplements typically contain 100–2,000 mg per capsule. By applying such dietary strategy, the recommended dietary allowance (RDA) of vitamin C could also be easily achieved, which is 90 mg/day for adult men and 75 mg/day for adult women (20). This again suggests the practicality and applicability of ascorbic acid as a dietary strategy for the prevention and treatment of hyperglycemia.

### Effect of Cooking

**Figure 2:**
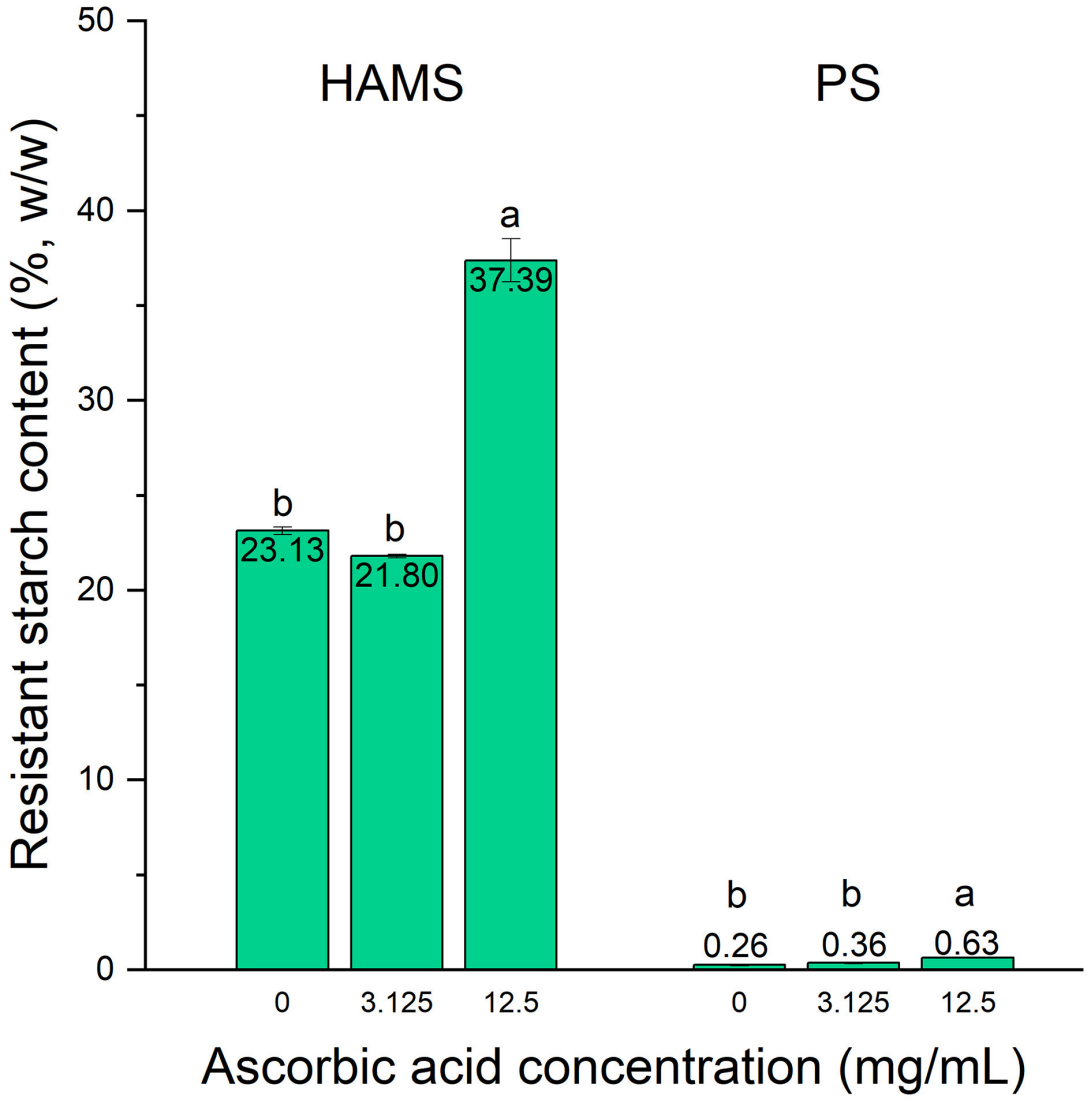
Resistant starch contents in high amylose maize starch (HAMS) and potato starch (PS), presented as a proportion of total starch, with the presence of ascorbic acid with concentrations of 3.125 and 12.5 mg/mL upon cooking. Error bars show standard deviation; *n* = 2. Significant differences among treatments are denoted by different letters (a > b, *p* < 0.05).

Starch digestibility is dependent on the activity of digestive enzymes as well as the characteristics of the starch itself, such as the botanical source and degree of gelatinization (21). Although the above results have confirmed the inhibitory effect of ascorbic acid on the *in vitro* digestion of raw starches, it is important to verify that such effect is present in forms of starch that would realistically be consumed in human diet, i.e., subjected to cooking. Accordingly, based on the results obtained from raw starches, we further studied the effects of ascorbic acid with concentrations of 3.125 and 12.5 mg/mL on the digestion of cooked HAMS and PS (Figure 2).

Heating starch in the presence of water, i.e., cooking, results in gelatinization of starch. This process causes the disruption and swelling of starch granules, leaching of amylose into the water, and the loss of molecular order in amylopectin. Upon heating, water enters the amorphous regions and then disrupts the crystalline regions. These changes are accompanied by swelling of the granules, which will contribute to the eventual collapse of the granules to form a paste if the water content is high enough (22). Consequently, the starch is more readily accessible and susceptible to enzymatic digestion (23). This outcome is reflected by the relatively low RS content for the cooked starches, which is 23.13 and 0.26% for HAMS and PS, respectively, as compared to 52.81 and 70.83% in their raw forms. PS was more susceptible to gelatinization as compared to HAMS, which could be due to its lower gelatinization temperature, which is 59–68°C as compared to 125°C for HAMS (24, 25). This result is consistent with other reports that the gelatinization of starch results in significant reductions of RS contents in canna, rice, and potato starches (5, 26, 27).

Similar to the results observed for raw starches, ascorbic acid with a concentration of 3.125 mg/mL did not present any inhibitory effect on starch digestion, while 12.5 mg/mL of ascorbic acid resulted in significant increases in the RS contents in both HAMS and PS, although the effects were rather modest as compared to those in raw starches. For cooked HAMS, the RS content was increased to 37.39% (*p* < 0.05) in the presence of ascorbic acid, whereas that of cooked PS was only increased to 0.63% (*p* < 0.05). Such modest enhancement could be explained by the destruction in the starch molecular packing order caused by gelatinization, which could not be recovered or altered in any way by the presence of ascorbic acid. This also indicates that ascorbic acid can only act on the digestive enzymes, but not the substrate, to resist enzymatic hydrolysis (11). Therefore, as the susceptibility of starch granules to enzymatic hydrolysis increases after cooking, fewer digestive enzymes are needed, resulting in the less potent inhibitory effect of ascorbic acid against starch digestion as compared to what was observed in raw starches. In addition, the modest effect could be attributed to the high moisture content of the cooking condition. After gelatinization, starch may undergo a process called retrogradation, in which the disordered amylose and amylopectin molecules re-associate into crystallized structures. Retrograded starch contributes to increased RS contents because the more ordered arrangement results in slower enzymatic digestion (28). The storage of gelatinized starch facilitates this process, but the water content plays a major role in the extent to which the starch retrogrades. More specifically, retrogradation for maize starch only occurs when the water content is between 20 and 90% (28). In our study, the moisture content of the gelatinized starch was ~97%, as the starches were suspended in the buffer solutions while being cooked, thereby rendering retrogradation unlikely. This could possibly lead to the low RS content after cooking even with the addition of ascorbic acid.

## Conclusion

The inhibitory effect of ascorbic acid on simulated *in vitro* starch digestion, as evidenced by increased contents of RS, was demonstrated in this study. Our results indicated the dose-dependent response of such inhibitory effect, and that the optimal level of ascorbic acid is practical and achievable via daily food or supplement intake and unlikely to result in toxicity in human body. Furthermore, in the tested cooking condition, the RS contents were significantly increased in the presence of ascorbic acid, although to a modest extent. This evidence suggests that ascorbic acid can be broadly applied to realistic starch systems. The inhibitory ability was most potent in the case of raw starch, and the slighter impact on cooked starch is likely the result of gelatinization with a high water content. Thus, future studies illustrating the inhibitory effect of ascorbic acid on starch cooked in relatively drier conditions are warranted to determine the effects of retrogradation on starch digestibility. Given that ascorbic acid has already been established as an essential nutrient, this study supports its further utilization as a promising agent in glycemic modulation.

## Data Availability Statement

The original contributions presented in the study are included in the article/supplementary material, further inquiries can be directed to the corresponding author.

## Author Contributions

AG: investigation, formal analysis, visualization, and writing—original draft. JG: investigation, formal analysis, visualization, and writing—review and editing. LT: conceptualization, formal analysis, and writing—review and editing. LK: conceptualization, supervision, formal analysis, visualization, and writing—review and editing. All authors contributed to the article and approved the submitted version.

## Conflict of Interest

The authors declare that the research was conducted in the absence of any commercial or financial relationships that could be construed as a potential conflict of interest.

## Publisher's Note

All claims expressed in this article are solely those of the authors and do not necessarily represent those of their affiliated organizations, or those of the publisher, the editors and the reviewers. Any product that may be evaluated in this article, or claim that may be made by its manufacturer, is not guaranteed or endorsed by the publisher.
